# The Effects of Anodal Transcranial Direct Current Stimulation on Sleep Time and Efficiency

**DOI:** 10.3389/fnhum.2020.00357

**Published:** 2020-08-27

**Authors:** Lindsey K. McIntire, R. Andy McKinley, Chuck Goodyear, John P. McIntire

**Affiliations:** ^1^Infoscitex, Inc., Wright-Patterson Air Force Base, Dayton, OH, United States; ^2^Air Force Research Laboratory/Applied Neuroscience Branch, Wright-Patterson Air Force Base, Dayton, OH, United States; ^3^Air Force Research Laboratory/Security and Intelligence Branch, Wright-Patterson Air Force Base, Dayton, OH, United States

**Keywords:** transcranial direct current stimulation, sleep time, sleep quality, sleep efficiency, sleep

## Abstract

A single session of anodal transcranial direct current stimulation (tDCS) has been shown to increase arousal in healthy participants for up to 24 h post-stimulation. However, little is known about the effects of tDCS on subsequent sleep in this population. Based on previous clinical studies, we hypothesized that anodal stimulation to the left dorsolateral prefrontal cortex (lDLPFC) would produce higher arousal with decreased sleep time and stimulation to the primary motor cortex (M1) would have the converse effect. Thirty-six active duty military were randomized into one of three groups (*n* = 12/group); active anodal tDCS over the lDLPFC, active anodal tDCS over left M1, or sham tDCS. Participants answered questionnaires 3 times a day and wore a wrist activity monitor (WAM) to measure sleep time and efficiency for 3 weeks. On weeks 2 and 3 (order counterbalance), participants received stimulation at 1800 h before 26 h of sustained wakefulness testing (sleep deprived) and at 1800 h without sleep deprivation (non-sleep deprived). There were no significant effects for the non-sleep deprived portion of testing. For the sleep deprived portion of testing, there were main effects of group and night on sleep time. The DLPFC group slept less than the other groups on the second and third night following stimulation. There is no negative effect on mood or sleep quality from a single dose of tDCS when participants have normal sleep patterns (i.e., non-sleep deprived portion of testing). The results suggest that stimulation may result in faster recovery from fatigue caused by acute periods of sleep deprivation, as their recovery sleep periods were less.

## Introduction

A unique non-invasive neuromodulation technology called transcranial direct current stimulation (tDCS) has shown efficacy for decades in clinical disorders such as major depressive disorder, Parkinson’s disease, stroke rehabilitation, schizophrenia, and chronic pain (Brunoni et al., 2012). In the past decade, there has been a rapid expansion of research into the cognitive benefits of tDCS on healthy populations (see Nitsche et al., 2009 and McKinley et al., 2012 for reviews). Researchers have shown enhancements in cognitive performance for tasks like navigation (Hampstead et al., 2014), visual search (Binney et al., 2018), and memory (Marshall et al., 2004; Gladwin et al., 2012; Javadi and Walsh, 2012), to name a few. Research from our own lab has shown that in military populations, anodal tDCS can improve learning and memory (McKinley et al., 2013), attention (Nelson et al., 2014), arousal (McIntire et al., 2017), and cognitive degradations due to sleep deprivation (McIntire et al., 2014, 2020). Furthermore, other military applications are being explored all over the world for cognitive, motor, and perceptual enhancement (see Davis and Smith, 2019 for review). However, little is known about the effects of tDCS on sleep time or sleep efficiency following a single exposure in these healthy populations. It is reasonable to suspect that tDCS could influence measures of subsequent sleep quality since tDCS has been found to effect arousal and attention in both sleep deprived and non-sleep deprived healthy participants. It is important to understand what if any side effects to sleep persist following stimulation in these populations for these purposes.

Previous studies on the enhancements in arousal, attention, and fatigue mitigation provided by tDCS in healthy participants delivered stimulation exclusively over the left dorsal lateral prefrontal cortex (lDLPFC) (McIntire et al., 2014, 2017). Research on the effects of tDCS on sleep has found that bi-frontal anodal stimulation right before bedtime does lower sleep time (Frase et al., 2016). Conversely, slow oscillatory tDCS over the frontal cortex during slow wave sleep has shown to increase slow-wave sleep immediately following stimulation (e.g., Eggert et al., 2013; Paßmann et al., 2016); however, some studies have shown an overall decrease in slow wave sleep as a result of the stimulation (Paßmann et al., 2016). Furthermore, behavioral benefits to memory and memory consolidation also appear largely age dependent with younger healthy adults experiencing an increase in memory consolidation but not older populations (Eggert et al., 2013; Paßmann et al., 2016). However, in the clinical literature, Roizenblatt et al. (2007) investigated sleep efficiency and arousal levels for patients with fibromyalgia. The authors found that anodal stimulation over the primary motor cortex (M1) increased sleep efficiency and decreased arousal while anodal stimulation of the dorsal lateral prefrontal cortex (DLPFC) decreased sleep efficiency and increased arousal. Further, recent research suggests that anodal tDCS to the DLPFC produces increased activity in the locus coeruleus (LC) region of the brain (Sherwood et al., 2018). The LC region is the primary norepinephrine nucleus and is believed to regulate attention, arousal, wakefulness, memory formation and memory retention which, are many of the behaviors enhanced in these studies. Other fMRI research demonstrated that anodal stimulation to the DLPFC increased profusion not only at the stimulation site but to regions anatomically connected, including M1 (Stagg et al., 2009). Because of these apparent behavioral trade-offs that seem dependent on stimulation site selection, it is unclear whether DLPFC or M1 is the ideal cortical target for fatigue reduction, or whether this varies between sleep deprived and non-sleep deprived individuals in healthy populations. It is also unclear if a single stimulation approximately 4 h prior to bedtime has any effect on sleep. Therefore, this study will examine the effects of one 30 min exposure of anodal tDCS over M1 and lDLPFC on sleep time and sleep efficiency, with and without sleep deprivation stress. We hypothesize that we may find differences in sleep time with DLPFC stimulation resulting in less sleep while M1 stimulation resulting in more sleep, similar to results seen in related clinical literature. We also anticipate that DLPFC stimulation will lead to increased subjective arousal when compared to the M1 location.

## Materials and Methods

### Equipment

#### tDCS Stimulator

The MagStim DC stimulator (Magstim Company Limited; Whiteland, United Kingdom) was used to provide the tDCS stimulation. This battery-powered device was controlled with a microprocessor to ensure constant current at up to 5,000 μA. For safety, multistage monitoring of the output current and electrode/tissue impedance is included. The device automatically shuts off if the impedance becomes greater than 50 kΩ to prevent electric shocks or burns. To allow for blinding, the device had numerical study codes that were pre-programmed to deliver either active or sham stimulation. An experimenter not involved with data collection supplied the codes to the research team.

#### tDCS Electrodes

The electrodes included an array of 5 electroencephalographic (EEG) electrodes arranged in a circular pattern purchased from Rio Grande Neurosciences (see Petree et al., 2011 for further details). Each electrode had an inner diameter of 1.6 cm yielding a contact area of 2.01 cm^2^ for each electrode. At 2 mA of supplied current, there was an average current density of 0.199 $\frac{\text{mA}}{{\text{cm}}^{2}}$. The anode electrode was placed either on the left dorsolateral prefrontal cortex (LDLPFC) or the left primary motor cortex (M1) depending on the participants’ randomly assigned condition. This corresponds to F3 and C3, respectively, using the International 10–20 System for electrode placement. The cathode was placed on the contralateral (right) upper bicep for both locations. The extracephalic reference was chosen to exclude effects due to the reference electrode (Nitsche et al., 2007). Electrode gel was used as the electrode to skin conduit. Sham stimulation consisted of a 15 s ramp up to the 2 mA current and a 15 s ramp down to mimic the skin sensation experienced during current ramp up.

#### Wrist Activity Monitor (WAM)

One week prior to any stimulation, participants’ were given a wrist activity monitor (WAM; AW64 Actiwatch, Ambulatory Monitoring, Inc.). The WAM was a non-invasive small electronic device that was worn on the wrist like a wristwatch for the entire duration of the study. It recorded limb and body movements through an accelerometer to determine when a participant was active and when they were asleep. The watch also measured ambient light and, in conjunction with the accelerometer data, was used to determine the amount of time the participants were asleep, and how efficient their sleep was based on the accelerometer data during the sleep cycle. Participants also recorded their approximate sleep and wake times to validate that the watch was working appropriately and ensure the correct sleep times were captured by the Actiware software. Compared to polysomnography, this device has been found to have high accuracy (86%), suggesting it is a reasonable device to measure sleep (see Marino et al., 2013 for a detailed study review).

### Subjects

Thirty-six active-duty military participants from Wright-Patterson Air Force Base completed this study. They were randomly assigned to one of three groups. There were 32 male and 4 female participants with an average age of 26 ± 5 years. Participants were compensated for their time but were disqualified if they met any of the exclusion criteria described in McKinley et al. (2012). In total, 50 participants enrolled in the study but 5 were dismissed because they met one or more of the exclusion criteria which, included: any neurological diagnosis, any psychological diagnosis/hospitalization, non-removable metal around the head, uncorrected vision impairments, sleep disorders, pregnant or trying to become pregnant, smoking, history of frequent headaches and/or migraines, history of seizures, history of fainting, high blood pressure or heart disease even if controlled with medication, and currently taking psychotropic or opioid medications. A further five participants withdrew prior to data collection due to work or family constraints on their time. And four participants self-withdrew at some point during the sleep deprivation data collection, reporting that they felt too tired to complete. Due to the self-withdraws during data collection, we finished the study with 13 participants receiving active stimulation over lDLPFC, 11 participants receiving active stimulation over M1, and 12 participants receiving sham stimulation.

### Subjective Questionnaire

Subjective affect was measured via the Visual Analog Scale (VAS) (Penetar et al., 1993). The VAS required participants to indicate the points on different lines that correspond to how he/she felt along the specified affect continuum at the time at which the test was taken (an analog subjective-magnitude rating scale with multiple scale items). The adjectives for the scale items included in the VAS were: Alert, Able to Concentrate, Anxious, Energetic, Feel Confident, Irritable, Jittery/Nervous, Sleepy, and Talkative. The indicated magnitudes were later measured and converted into numerical scores for analysis. The line is 10 cm long and the participant marks a tick on the line for each adjective on how they are feeling at that time. Scores are determined by measuring with a ruler where the tick is on the line. Participants filled the VAS out 3 times a day (AM, Noon, and PM) and then those scores from the day were averaged to make a single mood score for the day for each adjective.

### Procedures

Before any study procedures were carried out, the institutional review board on human research approved this study and written informed consent was obtained from all volunteers. Participants were randomly assigned to one of three groups for this placebo-controlled, double-blinded study. Group 1 (DLPFC) received anodal tDCS over the left DLPFC at 2 mA for 30 min, group 2 (M1) received anodal tDCS over the primary motor cortex at 2 mA for 30 min, and group 3 (sham) received 30 s of stimulation over lDLPFC.

After consent and screening, participants were given the WAM to wear for the duration of the study (3 weeks) and VAS questionnaires to fill out 3 times a day (AM, Noon, and PM) for the entire study. The first week served as baseline WAM and VAS measures. At the start of the 2nd and 3rd week participants received one 30 min session of stimulation according to their assigned condition at 1800 h. They then either participated in a 2 h cognitive testing block or a 26 h testing session under sleep deprivation stress. After both testing cases, participants were sent home for a week with the WAM and VAS. Therefore, WAM and VAS data consists of a week of baseline data, a week immediately following one 30 min dose without sleep deprivation stress (non-sleep deprived), and a week immediately following one 30 min dose with sleep deprivation stress (sleep deprived). The ordering of the weeks of non-fatigue vs. fatigue was counterbalanced within each group to avoid presentation order effects.

### Data Analysis

There were no significant differences found between the groups baseline data for any dependent variable (*p* > 0.05) using a one-way ANOVA. All other analyses used a change from baseline as the dependent variable. Sleep deprived and non-sleep deprived data was analyzed separately. The first model used was a mixed-repeated ANOVA with group a between factor (DLPFC, M1, Sham) and night as a within factor (1–5). Nights 4 and 5 were included in the model even though graphs indicated for the sleep deprived data, sleep time and efficiency returned to baseline on the 3^*rd*^ or 4^*th*^ night. Since the first 3 nights were of primary interest, a one-way ANOVA comparing groups was performed at each night even if a non-significant interaction (*p* > 0.05) was found between group and nights post-stimulation. *Post hoc* paired comparisons of group for a particular night used two-tailed two-sample *t*-tests with Cohen’s *d* used to determine effect size. If the variances were significantly different (*p* ≤ 0.05) then approximate *t*-test with Satterthwaite’s approximation for degrees of freedom was used. To test a mean change from baseline for each group, a two-tailed *t*-test was used. All comparisons used a per comparison error level of 0.05 with *p*-values provided. No correction for multiple comparisons was performed.

## Results

### Non-sleep Deprived Sleep Results

**FIGURE 1:**
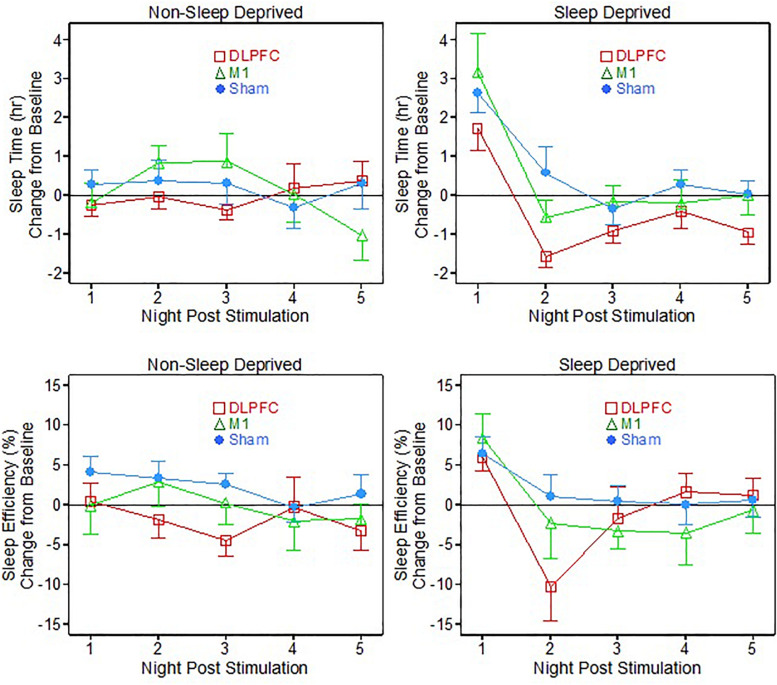
Sleep time and efficiency relative to baseline, following 1 dose of transcranial direct current stimulation (tDCS) with (Sleep deprived) and without (non-sleep deprived) sleep deprivation testing.

For the non-sleep deprived subjects, there was no significant main effect of neuromodulation group or post-stimulation night, nor a significant interaction effect of group and night, on measured sleep time or sleep efficiency (see Table 1 and Figure 1 – non-sleep deprived conditions).

**TABLE 1:** Wrist Activity Monitor(WAM) ANOVA results.

**Dependent variable**	**Condition**	**Effect**	**DF**	**DFe**	***F***	***p***
Sleep time	Non-sleep deprived	Group	2	31.15	0.21	0.809
		Night	4	121.97	0.63	0.645
		Group and night	8	121.93	1.26	0.271
	Sleep deprived	Group	2	29.31	3.87	0.032
		Night	4	111.43	25.04	<0.001
		Group and night	8	111.42	0.91	0.509
Sleep efficiency	Non-sleep deprived	Group	2	31.88	1.57	0.224
		Night	4	120.92	1.15	0.337
		Group and night	8	120.90	0.66	0.725
	Sleep deprived	Group	2	29.52	0.41	0.666
		Night	4	111.04	7.88	<0.001
		Group and night	8	111.03	1.68	0.111

### Sleep Deprived Sleep Results

For the sleep deprived portion of testing, there were some interesting significant results (see Table 1 and Figure 1– sleep deprived conditions). On the sleep time measure, there were significant main effects of both neuromodulation group [*F*(2, 29.3) = 3.87, *p* = 0.032] and post-stimulation night [*F*(4, 111.4) = 25.04, *p* < 0.001]. Specifically, *post hoc t*-testing showed that the DLPFC group slept significantly less time than both the M1 group (*p* = 0.045, Cohen’s *d* = 0.96) and the Sham group (*p* = 0.014, Cohen’s *d* = 1.11).

On the first night after sleep deprivation, all three groups slept significantly more than their own baselines: DLPFC (*t* = 3.04, *p* = 0.012), M1 (*t* = 3.08, *p* = 0.018), Sham (*t* = 5.29, *p* < 0.001). On the second night and thereafter following the sleep deprivation vigil, the M1 and Sham groups’ sleep times had returned to baseline. But surprisingly the DLPFC group had sleep times briefer than its own baseline average on Nights Two (*t* = -4.91, *p* < 0.001) and Three (*t* = -2.49, *p* = 0.032).

For the sleep efficiency metric, there was only a significant main effect for night of post-stimulation testing [*F*(4, 111.4) = 7.88, *p* < 0.001], with the groups all demonstrating higher sleep efficiency on the first night following the fatigue vigil, relative to their own baselines. On the second night post-vigil, the M1 and Sham groups had returned to baseline sleep efficiency, but the DLPFC group showed less sleep efficiency than its baseline (*t* = -2.35, *p* = 0.040).

A main effect for night of post-stimulation testing was expected for both sleep time and efficiency metrics for the sleep deprived participants, as all participants required significantly more sleep than usual on their first night after undergoing 36 h of continued wakefulness (this pattern is sometimes referred to “rebound” and/or “recovery” in the sleep deprivation or fatigue literatures; e.g., see Nakazawa et al., 1978).

**FIGURE 2:**
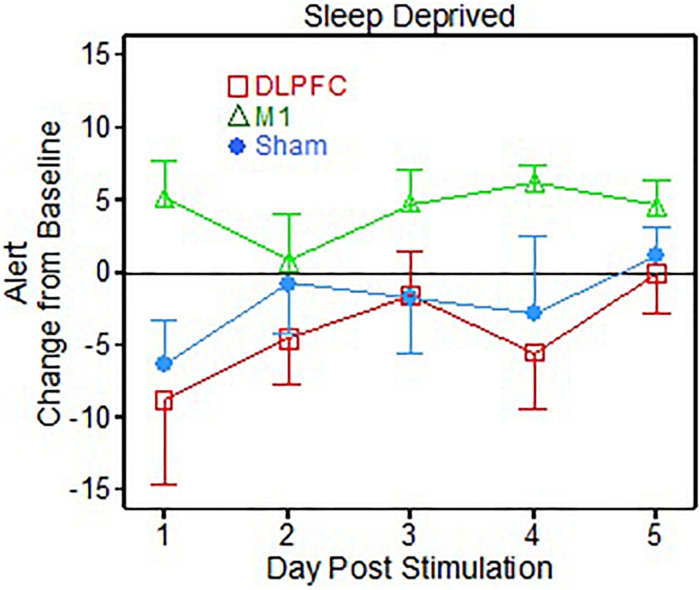
Visual Analog Scale (VAS) alert results following 1 dose of transcranial direct current stimulation (tDCS) for post-sleep deprivation testing (Sleep deprived).

For the VAS subject questionnaires, there were 9 scale items on the questionnaire. Of the 9 items there was one main effect found for group [*F*(2, 30.05) = 3.73, *p* = 0.036] for the “Alert” metric, corresponding to the sleep deprived portion of the testing (Figure 2). The *post hoc t*-tests showed that the M1 group reported feeling significantly more Alert than the DLPFC group (*p* = 0.012, Cohen’s *d* = 1.31), across post-stimulation days.

**FIGURE 3:**
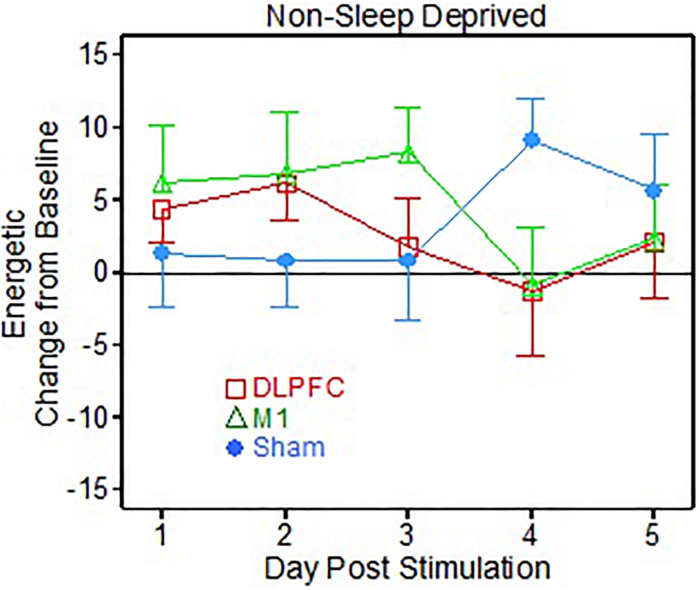
Visual Analog Scale (VAS) energetic results following 1 dose of transcranial direct current stimulation (tDCS) testing (Non-sleep deprived testing).

There was also a significant interaction effect of group × day [*F*(8, 120) = 2.51, *p* = 0.015] for the “Energetic” metric, corresponding to the non-sleep deprived portion of the testing (Figure 3). Specifically, the *post hoc t*-tests showed that the Sham group reported feeling more “energetic” on the VAS than the M1 group (*t* = -2.11, *p* = 0.049, Cohen’s *d* = 1.00), but only on the 4th day post-stimulation.

## Discussion

Over the past decade, research from our own lab and others have found that one 30 min dose of tDCS to the lDLPFC can improve arousal and sustained attention for as much as 24 h post-stimulation in healthy populations (McIntire et al., 2014, 2017; Nelson et al., 2014; McKinley et al., 2017). Stimulation location has been found to produce contrasting behavioral results throughout clinical and non-clinical populations. For example, a study on non-declarative memory formation in a healthy population found that cathodal tDCS over the DLPFC during memory consolidation and not stimulation over M1 during learning was the only montage that enhanced retention the next day compared to sham (McKinley et al., 2017). This study demonstrates that a single stimulation to the DLPFC affects performance at least 24 h after stimulation; therefore, it is possible that part of the behavioral results in this study could be the outcome of sleep quality differences between the time of stimulation and testing (one night of sleep). Clinical research has also found that arousal levels can be manipulated depending on location of the tDCS stimulation. For instance, in fibromyalgia patients it was discovered that stimulating the lDLPFC made patients feel more alert during the day but get less sleep at night, while stimulation to M1 had the opposite effect (Roizenblatt et al., 2007). While the research seems to be indicating that tDCS could be useful at mitigating performance declines associated with sleep loss and that the benefit of stimulation is long-lasting, little is known about the effects of stimulation on subsequent sleep quality. The aim of this study was to determine if a single 30 min dose of tDCS had an effect on subsequent sleep quality and if location of the stimulation altered those effects in a healthy population.

One of the primary findings for this study was that after the sleep deprivation vigil (sleep deprived participants), those receiving stimulation to the lDLPFC slept significantly less than the M1 and sham groups in the nights following sleep deprivation. Naturally, all groups slept more the first night immediately following the 36 h of continuous wakefulness, but the DLPFC group overall tended to sleep less than the other groups in the days after undergoing acute fatigue, potentially indicating faster recovery. No group reported feeling significantly more fatigued than any other group over the recovery time period, although on one subjective item out of 9, the M1 group reported significantly higher “alertness.” A meta-analysis comparing single to multiple (3–5 consecutive days) days of M1 stimulation in healthy individuals found significant motor learning improvements in both single and multiple stimulations compared to sham on memory retention suggesting long-lasting effects from a single stimulation (Hashermirad et al., 2016). This improved performance combined with improved alertness could indicate an improved sleep period the night following stimulation. In fact, tDCS applied during slow-wave sleep (SWS) has been found to increase the amount of SWS (e.g., Marshall et al., 2004; Eggert et al., 2013; Reato et al., 2013; Paßmann et al., 2016) as well as improve memory retention the next day (Marshall et al., 2004). However, all of these studies aimed at enhancing SWS to augment memory retention applied stimulation to the frontal cortex. Perhaps stimulation to the lDLPFC for treatment of sleep loss not only improves task-related performance during the sleep-loss period as evidenced in other sleep deprivation studies (e.g., McIntire et al., 2014, 2017, 2020, but also promotes faster recovery from fatigue, which would be consistent with this pattern of behavioral results and in line with Frase et al. (2016) that discovered bi-frontal anodal stimulation right before bedtime does lower sleep time Along these lines, previous researchers found that in a bipolar population, subjective sleep quality ratings significantly improved following 3 consecutive weeks of daily 20 min tDCS treatments to the lDLPFC (Minichino et al., 2014). Taken together, it is possible that both locations could promote faster recovery as evidenced by the objective and subjective findings across many studies discussed herein.

Contrary to our hypothesis, M1 stimulation did not result in higher subjective ratings of fatigue nor did the M1 group sleep more than the other groups. In fact, it was uncovered that the M1 group reported significantly higher alertness ratings than the DLPFC group in the days following sleep deprivation testing, possibly suggesting increased alertness/arousal for the M1 group. While some clinical research has found that stimulation to this area results in higher subjective fatigue ratings during the day (Roizenblatt et al., 2007), others have found the opposite effect. For example, in a post-polio syndrome population, it was uncovered that stimulation to this area for 15 days significantly improved subjective sleep quality and lessened fatigue symptoms (Acler et al., 2013). Therefore, it is possible that M1 also promotes faster recovery from fatigue. Research has found that a single session of M1 stimulation on healthy participants significantly improves memory retention the following day (Hashermirad et al., 2016; McKinley et al., 2017); indicating it could have an enhancement on sleep quality. It is also possible to have similar effects from the two anatomically close electrode locations due to the fact that tDCS is non-focal and the electrodes are also relatively large to allow for lower user discomfort (Miranda et al., 2006; Feira et al., 2009).

While most of our primary hypotheses were not supported by the data reported herein, overall, the pattern of results suggest that there is no noticeable, lingering negative effect on mood, sleep time, or sleep efficiency from a single 30 min dose of tDCS when participants have normal sleep patterns (i.e., when they are not undergoing lengthy sleep deprivation stress). The other results concerning sleep-deprived participants are mostly consistent with previous studies on sleep deprivation-induced fatigue; i.e., people sleep longer and more efficiently on the first day or two of recovery, as expected. Stimulation of the DLPFC might promote faster recovery from fatigue, as our data suggests that this group needed less sleep recovery time in the three nights immediately following a sleep deprivation vigil and is in line with previous work. It is also possible that M1 stimulation promotes faster recovery as they reported feeling higher levels of arousal than the other groups. This is consistent with behavioral findings in the research. Further research needs to examine the effects of both stimulation locations using polysomnography to verify these conclusions. It would also be important for future research to give cognitive testing after the sleep periods to assess any behavioral changes. Further, it could also be interesting to use a more focal form of tDCS called high-definition tDCS to determine if any differences in stimulation location exist in this context. Limitations of this study was the inability to exercise maximum control over sleep schedules and the exclusion of polysomnography to more precisely measure sleep quality. Other limitations include a small sample size and a higher male to female ratio due to our subject population (active-duty military). Future studies should also vary stimulation time to closer to bed time to see if stimulation interferes with sleep onset.

## Data Availability Statement

The raw data supporting the conclusions of this article are property of the United States Government and require clearance through Public Affairs before release. If clearance is given, they will be made available by the authors, without undue reservation, to any qualified researcher.

## Ethics Statement

The studies involving human participants were reviewed and approved by the AFRL IRB. The patients/participants provided their written informed consent to participate in this study.

## Author Contributions

LM: study design, data collection, and report writing. RM: study design and report writing. CG and JM: study design, data analysis, and report writing. All authors contributed to the article and approved the submitted version.

## Conflict of Interest

LM and CG were employed by Infoscitex, Inc. The remaining authors declare that the research was conducted in the absence of any commercial or financial relationships that could be construed as a potential conflict of interest.
